# Impact of Parental Socioeconomic Status on Childhood and Adolescent Overweight and Underweight in Korea

**DOI:** 10.2188/jea.JE20130056

**Published:** 2014-05-05

**Authors:** Jin-Won Noh, Young-eun Kim, Jumin Park, In-Hwan Oh, Young Dae Kwon

**Affiliations:** 1 Department of Healthcare Management, Eulji University, Seongnam, Korea; 2 Department of Biostatistics, Korea University College of Medicine, Seoul, Korea; 3 University of Maryland School of Nursing, Baltimore, MD, USA; 4 Department of Preventive Medicine, College of Medicine, Kyung Hee University, Seoul, Korea; 5 Department of Humanities and Social Medicine, College of Medicine and Catholic Institute for Healthcare Management, the Catholic University of Korea, Seoul, Korea

**Keywords:** childhood and adolescent overweight and underweight, Korea, parental factors, risk factor, socioeconomic status

## Abstract

**Background:**

The prevalence of overweight and underweight is steadily increasing among children and adolescents. To explore the relationship between parental socioeconomic status and body mass index, we examined levels of overweight and underweight among representative samples of children and adolescents in South Korea.

**Methods:**

We analyzed data from the 2009 Korean Survey on the Obesity of Youth and Children, conducted by the National Youth Policy Institute. The sample response rate for this survey was 93.9%. After excluding 745 subjects who had missing information on age, height, or weight, 9411 subjects were included. To measure parental socioeconomic status, 4 categories were assessed by using a structured questionnaire: subjective economic status, parental education level, parental occupational status, and family structure. We used the chi-squared test in univariable analysis and multinomial logistic regression in multivariable analysis.

**Results:**

Multinomial logistic regression analysis identified sex, education level, parental interest in weight management, and parental body shapes as statistically significant characteristics affecting overweight in children, and sex, place of residence, parental interest in weight management, and paternal and maternal body shapes as statistically significant characteristics affecting underweight (*P* < 0.05).

**Conclusions:**

Underweight and overweight coexist among adolescent Korean males of low socioeconomic status, which indicates that these conditions can coexist in developed countries. Appropriate interventions to address both overweight and underweight in adolescents are required.

## INTRODUCTION

Childhood and adolescence is a critical period of human life because it is characterized by rapid physical and sexual growth and changes in body fat that determine adult weight and height.^1^ Weight disorders such as overweight and underweight are now recognized as risk factors for health problems in childhood and adolescence.^2^

The prevalence of overweight is steadily increasing among children and adolescents. Previous studies showed that the rate of overweight in the United States had more than doubled in children and tripled in adolescents during the previous 30 years. In 2010, the prevalence of obesity among children and adolescents exceeded 30%.^3^^,^^4^ South Korea has also seen escalating overweight rates in children and adolescents, including a 70% increase from 1997 to 2005.^5^ The alarming increase in the prevalence of overweight may adversely affect physical and psychosocial health among children and adolescents and increase the risk of adult obesity.^6^

Due to standards of beauty portrayed by the mass media, underweight is as prevalent as overweight/obesity among children and adolescents, and girls are especially vulnerable.^7^ Previous studies revealed that underweight is linked to high rates of morbidity and mortality.^8^^,^^9^ We therefore attempted to identify critical factors influencing underweight and overweight among children and adolescents.

Increased understanding of the substantial influence of genetic, epigenetic, social, and environmental factors in childhood and adolescence has highlighted the importance of parental characteristics. Children living with overweight parents, especially mothers, are more likely to become overweight.^10^^,^^11^ The risk of childhood overweight is significantly increased when both parents are obese, as compared with children with 1 or no obese parents.^12^ However, sex differences in the relationship between parental weight status and childhood overweight remain controversial.^13^

It was reported that the risks of underweight and overweight in childhood and adolescence increase with parental socioeconomic status (SES). Since 1989 numerous studies have reported that childhood overweight is inversely correlated with other variables, including parental education, occupation, and income, which shows that lower parental SES is associated with less-healthful eating and more physical inactivity.^14^^,^^15^ In addition, studies have consistently found that the inverse correlation with overweight was stronger for parental education than for parental occupation or income.^14^ As with overweight, underweight may correlate with SES. Underweight was negatively associated with higher parental occupation and differed across regions.^16^ However, few studies have assessed the risk factors for underweight in school-aged children.

Although the associations of parental SES with childhood and adolescent thinness and overweight are known, research on these associations is limited. Previous studies of body weight among adolescents used unrepresentative samples and measured only conventional socioeconomic variables, which led to mixed results. In addition, most studies focused on overweight.^17^^–^^21^ Thus, a nationwide survey of both overweight and underweight is warranted. It is critical to identify additional modifiable socioeconomic risk factors to assist in developing programs that prevent and treat adolescent overweight.

We examined levels of overweight and underweight among representative samples of children and adolescents in South Korea and investigated the association between parental SES and body mass index (BMI).

## METHODS

### Data source and study samples

We analyzed data from the 2009 Korean Survey on the Obesity of Youth and Children, which was conducted by the National Youth Policy Institute (NYPI). This survey aimed to characterize the health status and lifestyle of children and adolescents and to use the data in future research and health policies for children and adolescents. The study included 10 156 children and adolescents (age 10–18 years) from 12 cities in Korea, excepting Jeju Island. Using procedures developed by the NYPI, the researchers selected a group of schools and trained interviewers visiting the schools to interview students using a structured questionnaire during the period from June through September in 2009; 93.9% of students agreed to participate in the interview.

After excluding 745 students with missing information on age, height, or weight, data from 9411 students were analyzed (5015 males, 4396 females; 856 underweight, 6797 normal weight, and 1758 obese). Informed consent was not required for this study because the analysis used data obtained from the 2009 Korean Survey on the Obesity of Youth and Children performed by the NYPI, which were completely de-identified and publicly released for research purposes. The present study was thus also exempt from full review by the ethical committee at Eulji University.

### Variables

BMI is commonly used to assess body fat composition and was defined as weight in kilograms divided by the height in meters squared (kg/m^2^). Various organizations, including the International Obesity Task Force (IOTF), World Health Organization, and US Centers for Disease Control and Prevention, have proposed BMI criteria for defining overweight and obesity in children and adolescents. In this study, participants were classified as underweight, normal weight, and overweight based on BMI values proposed by the Extended International IOTF (<18.5 kg/m^2^, 18.5 kg/m^2^ to 25 kg/m^2^, and >25 kg/m^2^, respectively). In this study, obese students (>30 kg/m^2^) were included in the overweight category.

The subjects had an average BMI of 19.9 kg/m^2^ (range 11.1–40.6 kg/m^2^) and an average weight of 51.1 kg (range 20.0–116.0 kg). The dependent variable in the analysis was weight status, which was classified as underweight, normal weight, and overweight.

We used a structured questionnaire to assess variables that affect weight status in children and adolescents. The explanatory variable, parental SES was measured by multiple factors. Four categories (subjective economic status, parental education level, parental occupational status, and family structure) were assessed to measure parental SES on the questionnaire. Participants were asked to rate their household economic status on a 7-point Likert scale (1 corresponded to “very poor” and 7 to “very wealthy”). We assessed paternal and maternal education level, which was classified as less than middle school, high school, college, and graduate school. Parental occupational status was set as employed or unemployed, without specifying the type of occupation. Family structure was classified as parents living with children, single-parent family (living with a single father or single mother), and non-parental guardian. Participants were asked to rate parental interest in their weight management on a 5-point Likert scale (1 corresponding to “very interested” and 5 corresponding to “no interest”). In addition, subjective parental weight status was assessed using a 5-point Likert scale ranging from very slim (1) to very heavy (5).

### Statistical analysis

The chi-squared test was used for univariable analysis. Because the outcome variable was classified into 3 categories, we performed multinomial logistic regression to estimate odds ratios (ORs) with 95% CIs, with normal weight as the reference category. We also compared non-overweight (underweight plus normal) with overweight students in sensitivity analysis. We used backward elimination with a significance level of 0.05 to select variables in this study. The variance inflation factor (VIF) was calculated to assess multicollinearity in the logistic regression model. We used SAS statistical software, version 9.2 (SAS Institute, Cary, NC, USA), for all data analyses.

## RESULTS

### General characteristics of subjects

Of the 9411 enrolled participants, 5015 (53.3%) were male and 4396 (46.7%) were female; 29.2% were elementary school students and 70.8% were senior secondary school students. As for economic status, 19.7% participants described their economic status as low, 46.1% as average, and 34.3% as high. Most students (72.2%) were of normal weight, 18.7% were overweight, and 9.1% were underweight. The average (SD) BMI was 19.9 (3.3). Among students in single-parent households (8.8%), 345 (3.7%) students were living with their father and 481 (5.1%) were living with their mother. The remaining 486 (5.2%) students lived with grandparents, relatives, and/or siblings (Table 1).

**Table 1.** Demographic characteristics of children and adolescents and bivariate associations between covariates and body mass index category

Variable	Category	Total (*N* = 9411) (*n*, %) Mean ± SD (min, max)	Body Mass Index		

			Underweight (*N* = 856) (*n*, %)	Normal (*N* = 6797) (*n*, %)	Overweight (*N* = 1758) (*n*, %)
Body mass index		19.9 ± 3.3 (11.1, 40.6)	856 (9.1)	6797 (72.2)	1758 (18.7)
Sex*	Male	5015 (53.3)	309 (6.2)	3482 (69.4)	1224 (24.4)
	Female	4396 (46.7)	547 (12.4)	3315 (75.4)	534 (12.2)
Education*	Elementary school, grades 4 to 6	2745 (29.2)	221 (8.1)	1847 (67.3)	677 (24.7)
	Senior secondary school	6666 (70.8)	635 (9.5)	4950 (74.3)	1081 (16.2)
Region	Capital city (Seoul)	1568 (16.8)	151 (9.6)	1125 (71.8)	292 (18.6)
	Metropolitan city	3090 (33.1)	252 (8.2)	2249 (72.8)	589 (19.1)
	Smaller cities and countryside	4675 (50.1)	449 (9.6)	3362 (71.9)	864 (18.5)
Economic status	Low	1788 (19.7)	175 (9.8)	1267 (70.9)	346 (19.4)
	Average	4187 (46.1)	397 (9.5)	3062 (73.1)	728 (17.4)
	High	3115 (34.3)	252 (8.1)	2248 (72.2)	615 (19.7)
Paternal education	Middle school graduate or lower	465 (5.2)	40 (8.6)	330 (71.0)	95 (20.4)
	High school graduate	3743 (41.6)	342 (9.1)	2708 (72.4)	693 (18.5)
	University graduate or higher	4780 (53.2)	435 (9.1)	3472 (72.6)	873 (18.3)
Maternal education	Middle school graduate or lower	475 (5.3)	32 (6.7)	349 (73.5)	94 (19.8)
	High school graduate	4700 (52.4)	431 (9.2)	3410 (72.6)	859 (18.3)
	University graduate or higher	3795 (42.3)	346 (9.1)	2726 (71.8)	723 (19.1)
Father working?	No	349 (3.8)	26 (7.5)	244 (69.9)	79 (22.6)
	Yes	8801 (96.2)	803 (9.1)	6378 (72.5)	1620 (18.4)
Mother working?	No	3090 (33.7)	293 (9.5)	2236 (72.4)	561 (18.2)
	Yes	6069 (66.3)	531 (8.8)	4392 (72.4)	1146 (18.9)
Parental interest in weight control*	Very high	3874 (41.3)	360 (9.3)	2449 (63.2)	1065 (27.5)
	Average	3432 (36.6)	282 (8.2)	2607 (76.0)	543 (15.8)
	Little	2069 (22.1)	213 (10.3)	1712 (82.8)	144 (7.0)
Paternal body shape*	Slim	2169 (23.2)	269 (12.4)	1551 (71.5)	349 (16.1)
	Average	4077 (43.6)	353 (8.7)	3008 (73.8)	716 (17.6)
	Obese	3106 (33.2)	232 (7.5)	2201 (70.9)	673 (21.7)
Maternal body shape*	Slim	2069 (22.1)	235 (11.4)	1450 (70.1)	384 (18.6)
	Average	4159 (44.4)	383 (9.2)	3057 (73.5)	719 (17.3)
	Obese	3130 (33.4)	233 (7.4)	2253 (72.0)	644 (20.6)
Family structure	Parents living with children	8099 (86.1)	737 (9.1)	5868 (72.5)	1494 (18.5)
	Single-parent family (father)	345 (3.7)	37 (10.7)	251 (72.8)	57 (16.5)
	Single-parent family (mother)	481 (5.1)	38 (7.9)	335 (69.7)	108 (22.5)
	Non-parent	486 (5.2)	44 (9.1)	343 (70.9)	99 (20.4)

*P < 0.1 based on chi-squared test for the bivariate association of covariates and body mass index category.

### General characteristics of underweight, normal-weight, and overweight students

We performed univariable analysis to identify differences in individual characteristics that were associated with overweight. Males were more obese than females (*P* < 0.001), and students in the fourth to sixth grade of elementary school were more overweight than secondary school students (*P* < 0.001). Children living with overweight parents were more overweight (paternal body shape, *P* < 0.001; maternal body shape, *P* < 0.001). Children’s weight status was positively associated with the degree of parental interest in weight management (*P* < 0.001). Although children from both low and high economic status were more likely to be overweight than were those of average economic status, the results were not statistically significant (*P* = 0.2) (Table 1).

### Effects of parental SES on overweight in children

Univariable analysis showed that sex, educational background, parental economic status, paternal employment status, parental interest in weight management, and paternal and maternal body shape significantly differed in the overweight group as compared with the normal-weight group (*P* < 0.05). Although place of residence, parental education level, and parental employment status were not significant variables in univariable analysis, they are often used as proxy measures of economic status in South Korea and serve as independent variables. Presence of parents in the student’s household and parental education level were also independent variables in this study and were adjusted for in the analysis (Table 2).

**Table 2.** Crude odds ratios (ORs) and 95% CIs from multinomial logistic regression of the likelihood of being underweight and overweight (vs normal weight)

Variable	Category	Underweight^a^		Overweight^a^	
	
		OR (95% CI)		OR (95% CI)	
Sex	Male	1.0		1.0	
	Female	1.9	(1.6, 2.2)*	0.5	(0.4, 0.5)*
Education	Elementary school, grades 4 to 6	0.9	(0.8, 1.1)	1.8	(1.6, 2.0)*
	Senior secondary school	1.0		1.0	
Region	Capital city (Seoul)	1.0	(0.8, 1.2)	1.0	(0.9, 1.2)
	Metropolitan city	0.8	(0.7, 0.9)*	1.0	(0.9, 1.2)
	Smaller cities and countryside	1.0		1.0	
Economic status	Low	1.2	(1.0, 1.5)	1.0	(0.8, 1.1)
	Average	1.1	(0.9, 1.3)	0.9	(0.8, 1.0)*
	High	1.0		1.0	
Paternal education	Middle school graduate or lower	1.0	(0.7, 1.4)	1.2	(0.9, 1.5)
	High school graduate	1.0	(0.9, 1.2)	1.0	(0.9, 1.1)
	University graduate or higher	1.0		1.0	
Maternal education	Middle school graduate or lower	1.0		1.0	
	High school graduate	1.3	(0.9, 1.9)	0.9	(0.7, 1.1)
	University graduate or higher	1.3	(0.9, 1.9)	1.1	(1.0, 1.2)
Father working?	No	0.8	(0.5, 1.3)	1.3	(1.0, 1.7)*
	Yes	1.0		1.0	
Mother working?	No	1.1	(0.9, 1.3)	1.0	(0.9, 1.1)
	Yes	1.0		1.0	
Parental interest in weight control	Very much	1.2	(1.0, 1.4)*	5.1	(4.2, 6.2)*
	Average	0.8	(0.7, 1.0)	2.4	(1.9, 2.9)*
	Little	1.0		1.0	
Paternal body shape	Slim	1.6	(1.3, 2.0)*	0.7	(0.6, 0.9)*
	Average	1.1	(0.9, 1.4)	0.8	(0.7, 0.9)*
	Obese	1.0		1.0	
Maternal body shape	Slim	1.6	(1.3, 2.0)*	0.9	(0.8, 1.1)
	Average	1.3	(1.1, 1.5)*	0.8	(0.7, 0.9)*
	Obese	1.0		1.0	
Family structure	Parents living with children	1.0		1.0	
	Single-parent family (father)	1.3	(0.8, 1.9)	0.9	(0.6, 1.2)
	Single-parent family (mother)	1.0	(0.7, 1.5)	1.1	(0.9, 1.5)
	Non-parent	1.0	(0.7, 1.5)	1.1	(0.9, 1.4)

^a^The reference category in the multinomial logistic model is those with normal weight.

*P < 0.05.

Multivariable multinomial logistic regression analysis showed that sex, education level, parental interest in weight management, and parental body shapes were significantly associated with overweight in children (*P* < 0.05). The effect of parental economic status on overweight among their children was slightly greater for lower levels of parental economic status of parents (low vs high: OR = 1.2, 95% CI 1.0–1.4); however, the result was not statistically significant (Table 3).

**Table 3.** Adjusted odds ratios (ORs) and 95% CIs from multinomial logistic regression of the likelihood of being underweight and overweight (vs normal weight)

Variable	Category	Underweight^a^		Overweight^a^	
	
		OR (95% CI)		OR (95% CI)	
Sex	Male	1.0		1.0	
	Female	1.8	(1.6, 2.1)*	0.4	(0.3, 0.4)*
Education	Elementary school, grades 4 to 6	0.9	(0.7, 1.1)	1.8	(1.6, 2.1)*
	Senior secondary school	1.0		1.0	
Region	Capital city (Seoul)	0.9	(0.8, 1.2)	1.0	(0.8, 1.2)
	Metropolitan city	0.8	(0.7, 0.9)*	1.1	(0.9, 1.2)
	Smaller cities and countryside	1.0		1.0	
Economic status	Low	1.2	(1.0, 1.6)	1.2	(1.0, 1.4)
	Average	1.1	(0.9, 1.3)	1.1	(0.9, 1.2)
	High	1.0		1.0	
Paternal education	Middle school graduate or lower	1.2	(0.7, 1.8)	1.3	(0.9, 1.8)
	High school graduate	1.0	(0.9, 1.3)	1.1	(0.9, 1.3)
	University graduate or higher	1.0		1.0	
Maternal education	Middle school graduate or lower	1.0		1.0	
	High school graduate	1.4	(0.9, 2.2)	0.9	(0.7, 1.2)
	University graduate or higher	1.5	(0.9, 2.3)	0.9	(0.7, 1.3)
Father working?	No	0.8	(0.5, 1.2)	1.3	(1.0, 1.8)
	Yes	1.0		1.0	
Mother working?	No	1.1	(1.0, 1.3)	1.0	(0.9, 1.1)
	Yes	1.0		1.0	
Parental interest in weight control	Very much	1.0	(0.9, 1.3)	5.9	(4.9, 7.3)*
	Average	0.8	(0.6, 1.0)*	2.5	(2.1, 3.1)*
	Little	1.0		1.0	
Paternal body shape	Slim	1.6	(1.3, 2.0)*	0.8	(0.7, 0.9)*
	Average	1.1	(0.9, 1.4)	0.8	(0.7, 0.9)*
	Obese	1.0		1.0	
Maternal body shape	Slim	1.7	(1.4, 2.1)*	0.9	(0.7, 1.0)
	Average	1.4	(1.2, 1.7)*	0.7	(0.6, 0.8)*
	Obese	1.0		1.0	

^a^The reference category in the multinomial logistic model is those with normal weight.

*P < 0.05.

Using background elimination the variables sex, education background, paternal employment status, parental interest in weight management, and paternal and maternal body shape were selected as significant. Education background, parental economic status, parental interest in weight management, and maternal body shape were selected as significant variables in the analysis of male students. Education background, maternal education background, parental interest in weight management, and paternal and maternal body shape were significant for female students (Table 4).

**Table 4.** Adjusted odds ratio (ORs) and 95% CIs from multinomial logistic regression (backward elimination) of the likelihood of being underweight and overweight (vs normal weight) by sex

Variable	Category	Total				Male				Female			
		
		Underweight^a^		Overweight^a^		Underweight^a^		Overweight^a^		Underweight^a^		Overweight^a^	
					
		OR (95% CI)		OR (95% CI)		OR (95% CI)		OR (95% CI)		OR (95% CI)		OR (95% CI)	
Sex	Male	1.0		1.0									
	Female	1.8	(1.6, 2.2)*	0.4	(0.3, 0.4)*								
Education	Elementary school, grades 4 to 6	0.9	(0.7, 1.1)	1.8	(1.6, 2.0)*	1.0	(0.7, 1.4)	1.5	(1.3, 1.8)*	0.9	(0.7, 1.1)	2.5	(2.0, 3.1)*
	Senior secondary school	1.0		1.0		1.0		1.0		1.0		1.0	
Region	Capital city (Seoul)	1.0	(0.8, 1.2)	1.0	(0.8, 1.2)								
	Metropolitan city	0.8	(0.7, 0.9)*	1.1	(1.0, 1.2)								
	Smaller cities and countryside	1.0		1.0									
Economic status	Low					1.8	(1.3, 2.5)*	1.2	(1.0, 1.5)				
	Average					1.2	(0.9, 1.6)	1.2	(1.0, 1.4)*				
	High					1.0		1.0					
Paternal education	Middle school graduate or lower												
	High school graduate												
	University graduate or higher												
Maternal education	Middle school graduate or lower									1.6	(0.9, 2.7)	0.6	(0.4, 0.9)*
	High school graduate									1.5	(0.9, 2.5)	0.5	(0.3, 0.8)*
	University graduate or higher									1.0		1.0	
Father working?	No	0.8	(0.5, 1.3)	1.4	(1.1, 1.9)*	0.4	(0.2, 1.0)	1.3	(0.9, 1.9)				
	Yes	1.0		1.0		1.0		1.0					
Mother working?	No												
	Yes												
Parental interest in weight control	Very much	1.0	(0.9, 1.3)	5.9	(4.8, 7.2)*	2.2	(1.6, 3.0)*	6.2	(5.0, 7.8)*	0.7	(0.5, 0.8)*	5.7	(3.7, 8.7)*
	Average	0.8	(0.6, 1.0)*	2.5	(2.0, 3.1)*	1.2	(0.9, 1.7)	2.7	(2.1, 3.4)*	0.6	(0.5, 0.8)*	2.2	(1.4, 3.4)*
	Little	1.0		1.0		1.0		1.0		1.0		1.0	
Paternal body shape	Slim	1.6	(1.3, 2.0)*	0.8	(0.7, 0.9)*	1.5	(1.1, 2.2)*	0.9	(0.8, 1.1)	1.6	(1.3, 2.1)*	0.6	(0.5, 0.8)*
	Average	1.1	(0.9, 1.4)	0.8	(0.7, 0.9)*	1.1	(0.8, 1.5)	0.8	(0.7, 1.0)*	1.1	(0.9, 1.4)	0.6	(0.5, 0.8)*
	Obese	1.0		1.0		1.0		1.0		1.0		1.0	
Maternal body shape	Slim	1.7	(1.4, 2.1)*	0.8	(0.7, 1.0)*	1.6	(1.1, 2.3)*	1.0	(0.8, 1.2)	1.9	(1.4, 2.4)*	0.7	(0.5, 0.9)*
	Average	1.4	(1.2, 1.7)*	0.7	(0.6, 0.8)*	1.4	(1.0, 1.9)*	0.7	(0.6, 0.9)*	1.4	(1.1, 1.8)*	0.7	(0.5, 0.9)*
	Obese	1.0		1.0		1.0		1.0		1.0		1.0	

^a^The reference category in the multinomial logistic model is those with normal weight.

*P < 0.05.

We used logistic regression in a sensitivity analysis of non-overweight (underweight, normal) versus overweight children. Sex, education level, paternal education level, paternal employment status, parental interest in weight management, and paternal and maternal body shape were statistically significant (Table 5).

**Table 5.** Adjusted odds ratios (ORs) and 95% CIs from logistic regression (backward elimination) of the likelihood of being over underweight (vs underweight) and over normal (vs underweight or normal)

Variable	Category	(Normal, Overweight) vs Underweight		Overweight vs (Underweight, Normal)	
	
		OR (95% CI)		OR (95% CI)	
Sex	Male	1.0		1.0	
	Female	0.5	(0.4, 0.5)*	0.3	(0.3, 0.4)*
Education	Elementary school, grades 4 to 6	1.3	(1.1, 1.5)*	1.8	(1.6, 2.1)*
	Senior secondary school	1.0		1.0	
Region	Capital city (Seoul)	1.0	(0.9, 1.3)		
	Metropolitan city	1.3	(1.1, 1.5)*		
	Smaller cities and countryside	1.0			
Economic status	Low				
	Average				
	High				
Paternal education	Middle school graduate or lower			1.4	(1.1, 1.8)*
	High school graduate			1.1	(1.0, 1.3)
	University graduate or higher			1.0	
Maternal education	Middle school graduate or lower				
	High school graduate				
	University graduate or higher				
Father working?	No			1.4	(1.0, 1.9)*
	Yes			1.0	
Mother working?	No				
	Yes				
Parental interest in weight control	Very much	1.2	(1.0, 1.5)*	5.9	(4.8, 7.1)*
	Average	1.4	(1.1, 1.7)*	2.6	(2.1, 3.2)*
	Little	1.0		1.0	
Paternal body shape	Slim	0.6	(0.5, 0.7)*	0.7	(0.6, 0.9)*
	Average	0.8	(0.7, 1.0)	0.8	(0.7, 0.9)*
	Obese	1.0		1.0	
Maternal body shape	Slim	0.6	(0.5, 0.7)*	0.8	(0.7, 1.0)*
	Average	0.7	(0.6, 0.8)*	0.7	(0.6, 0.8)*
	Obese	1.0		1.0	

*P < 0.05.

### Effects of parental SES on underweight in students

Sex, place of residence, parental interest in weight management, and paternal and maternal body shape were significant (*P* < 0.05) in univariable analysis comparing normal-weight and underweight children.

In multivariable logistic regression analysis of the effects of underweight in students, the analyzed variables were identical to those included in the univariable analysis of normal-weight and overweight students. In the analysis, sex, place of residence, parental interest in weight management, and paternal and maternal body shape were statistically significant. The effect of parental economic status on underweight in their children slightly increased at lower levels of parental economic status (low vs high: OR = 1.2, 95% CI 1.0–1.6), but the result was not statistically significant (Table 3).

Sex, place of residence, parental interest in weight management, and paternal and maternal body shape were identified as significant variables using backward elimination. For males, the significant variables identified using backward elimination were parental economic level, parental interest in weight management, and maternal body shape. For females, the significant variables were parental interest in weight management and parental body shape (Table 4).

In sensitivity analysis between non-underweight (normal, overweight) and underweight children, sex, education level, region, parental interest in weight management, and paternal and maternal body shape were statistically significant (Table 5).

## DISCUSSION

We used data from the 2009 NYPI Survey to identify risk factors for high and low BMI in children and adolescents. The weight status of children and adolescents was classified as underweight, normal weight, and overweight. Multinomial logistic regression was used to identify the risk factors. Regarding BMI status, 9.1% of students were underweight, 72.2% were normal weight, and 18.7% were overweight. The proportion of underweight in females was double that in males. In contrast, the proportion of overweight in males was double that in females. In multinomial logistic regression, low economic status was a statistically significant SES risk factor in males. Similarly, high economic status was a protective factor for overweight. Among females, low maternal education level was associated with the risk of overweight. In addition, parental body shape was strongly associated with underweight and overweight in their children. In male adolescents, parental body shape was related to BMI. As compared with the children of obese mothers, children of slim or average-weight mothers had a higher risk of underweight. As compared with the children of average-weight mothers, the risk of overweight was higher for children with slim and obese mothers. The effects of body shape were similar for female adolescents.

Concerns regarding the health effects of overweight have increased throughout the world because of the rapid increase in overweight.^3^^–^^5^ In addition, the importance of the effect of parental SES on overweight among their children is widely recognized.^17^^–^^19^ Studies have examined the relationship between SES and overweight in Korean adolescents.^20^^–^^22^ However, adolescent underweight is much less studied in Korea, even though it can result in a substantial disease burden.^8^^,^^9^ For this reason, studies of the factors leading to underweight in childhood and adolescence are needed. To our knowledge, the association of SES with underweight in children and adolescents has not been previously investigated in the Korean population.

In this study, risk factors for underweight and overweight among Korean adolescents were identified by multinomial logistic regression analysis of nationally representative data. The results show that both underweight and overweight are associated with low parental SES in male adolescents, although fewer students were underweight than overweight.

Although previous studies noted a positive correlation between overweight and SES in developing countries, an inverse correlation was reported in developed countries, particularly in women.^23^ With nutritional transition and economic growth, the relationship between SES and overweight changes, and the burden of overweight is borne by those with low SES. Simultaneously, underweight due to malnutrition is an important concern among those with low SES. This has been referred to as the double nutrition burden.^24^^,^^25^ Monterio et al concluded that low-income Brazilian women were more likely to be either underweight or overweight than women with higher incomes. Some evidence suggests that this is also the case among children and adolescents.^16^^,^^26^ In Scottish preschool children, the OR for underweight in the most deprived group was 1.51 versus the least deprived groups. Regarding overweight, the OR was 1.30 in the most deprived group. In a German study of children and adolescents aged 11 to 17 years, low parental occupation was associated with the risk of underweight. In contrast, low family affluence was related to the risk of overweight.^16^ Our results suggest that the problems of underweight and overweight coexist in Korean adolescent males of low SES. The overweight pattern found in other developed countries was also seen among South Korean women. A previous study of adolescents found an inverse relationship between SES and overweight.^22^ In the present study, underweight and overweight were more prevalent among male adolescents of low SES, which indicates that these conditions can coexist in developed countries. Therefore, underweight is a concern even in developed countries. Thus, interventions should address both underweight and overweight in childhood and adolescence.

It should be noted that underweight and overweight only coexisted among males. Regarding underweight, the mass media promotes slimness as the standard of beauty. Some studies found that the prevalence of underweight and overweight might be comparable in young people, especially among girls.^7^ In Korean society, attitudes toward those who are overweight or obese tend to be negative. More than 70% of female adolescents of normal weight perceive themselves as overweight and try to lose weight.^27^ Another study found that female students with high SES were more likely to have a lower BMI.^28^ We hypothesize that the cultural preference for slimness affects females of high SES and that underweight and overweight do not coexist, because female adolescents of high SES are much more likely to be underweight. The cultural preference for slimness does not extend to males; therefore, underweight males are more likely to have low SES.

Parental body shape was strongly associated with underweight and overweight, which could be due to genetics and/or family lifestyle. For example, when parents were slim, the possibility of underweight significantly increased among their children. In addition, the possibility of overweight increased when maternal body shape was obese. Among obese children, the effect of paternal body shape was less clear than that of maternal body shape. Because data on parental body shape were collected from the survey, bias is possible. For example, a slim maternal body shape was associated with overweight in male adolescents.^7^ Future studies should investigate the effects of parental body shape on their children’s BMI.

This study has some limitations. The cross-sectional study design does not allow analysis of cause-and-effect relationships. For example, parental interest in weight control was strongly associated with underweight and overweight. This interest could be the result of weight status rather than the cause. A cohort study could overcome this limitation and could yield stronger evidence of a relation between SES and weight. In addition, other possibly related or important factors were not considered in this study. For example, because a family affluence scale was not used, comparisons with findings from other countries is not straightforward. Additionally, although parental body shape was used as a measure of genetic and lifestyle factors, the use of self-administered questionnaires did not allow us to collect data on parental BMI.

Despite these limitations, this study has important implications. First, this study used nationally representative Korean data to examine the effects of risk factors on BMI during childhood and adolescence. Second, the results confirm that, although underweight was less prevalent than overweight, it is indeed a concern in developed countries. Therefore, appropriate interventions should be designed to address malnutrition. In addition, sex differences in overweight patterns, including the effects of cultural preferences, need to be considered in any attempt to address the problem of coexisting underweight and overweight. Indeed, it might be possible to measure the effects of genetic and lifestyle factors if an appropriate variable can be identified and used in future research.

### Conclusions

We noted the coexistence of underweight and overweight among male children and adolescents of low SES. In female adolescents, an inverse relationship was observed between overweight and SES, as determined by maternal education level. Our finding of an inverse relationship with overweight confirms the results of a previous study of Korean adolescents. With respect to underweight, our results are similar to those of a German and Scottish study and imply that underweight and overweight can coexist in a developed country.
